# Efficacy of Tegoprazan in Patients with Laryngopharyngeal Reflux Disease: A Preliminary Feasibility Study

**DOI:** 10.3390/jcm12196116

**Published:** 2023-09-22

**Authors:** Hye Kyung Jeon, Gwang Ha Kim, Yong-Il Cheon, Sung-Chan Shin, Byung Joo Lee

**Affiliations:** 1 Department of Internal Medicine, Pusan National University School of Medicine, Busan 49241, Republic of Korea; kyung3842@hanmail.net; 2 Biomedical Research Institute, Pusan National University Hospital, Busan 49241, Republic of Korea; 3 Department of Otolaryngology, Pusan National University School of Medicine, Busan 49241, Republic of Korea; skydragonone@naver.com (Y.-I.C.); shinsc0810@gmail.com (S.-C.S.)

**Keywords:** laryngopharyngeal reflux disease, potassium-competitive acid blocker, tegoprazan, treatment effectiveness

## Abstract

Tegoprazan is a novel, potent, and highly selective potassium-competitive acid blocker that inhibits gastric acid secretion with rapid onset of action and prolonged control of gastric acidity. We performed a preliminary feasibility study to evaluate whether tegoprazan could control symptoms more effectively than a placebo in patients with laryngopharyngeal reflux disease (LPRD). In this double-blind, randomized, placebo-controlled trial, 35 patients with LPRD were randomly assigned to two groups: tegoprazan 50 mg daily and placebo. The primary endpoint was the complete resolution rate of LPRD symptoms after 8 weeks of medication, and the secondary endpoints were the complete resolution rate of LPRD symptoms after 4 weeks of medication and changes in the reflux symptom index (RSI) and reflux finding score (RFS) from baseline at 4 and 8 weeks of medication. There was no difference in the complete symptom resolution rates at 8 weeks between the tegoprazan and placebo groups (29.4% [5/17] vs. 27.8% [5/18], *p* = 1.000). Moreover, there was no significant difference in the complete symptom resolution rates at 4 weeks between the two groups. Compared with the baseline, both tegoprazan and placebo significantly reduced the total RSI and RFS scores after 4 and 8 weeks of medication; however, tegoprazan was not superior to the placebo. In conclusion, tegoprazan (50 mg daily) administration improved LPRD symptoms and signs. However, tegoprazan did not show superiority over placebo. Considering the potential effectiveness of tegoprazan as an acid-suppressing therapy and the possibility of type II error due to a low number of included patients herein, prospective, large-scale, multi-center studies with a higher dose of tegoprazan for a prolonged duration are required to elucidate the efficacy of tegoprazan in patients with LPRD. (ClinicalTrials.gov: NCT05871398).

## 1. Introduction

Laryngopharyngeal reflux disease (LPRD) is defined as several symptoms and signs caused by the retrograde flow of gastroduodenal contents into the laryngopharynx, oropharynx, and nasopharynx [1]. LPRD symptoms include hoarseness, throat clearing, sore throat, a globus sensation, chronic cough, dysphagia, and postnasal drip, and the endolaryngeal signs of LPRD include erythema, edema, and interarytenoid hypertrophy [2]. To define LPRD, self-administered tools such as the reflux symptom index (RSI) [3] and reflux finding score (RFS) [4], based on endolaryngeal signs, are clinically used.

The pathogenesis and treatment of LPRD remain controversial, and most clinicians believe that LPRD is an extraesophageal variant of gastroesophageal reflux disease (GERD) [5,6]. There are two main theories on LPRD related to acid reflux: the reflux and reflex theories. The reflux theory states that gastric acid and pepsin damage the larynx directly via the reflux of gastric contents [2], and the reflex theory states that acid reflux in the distal esophagus stimulates vagal-mediated reflexes, leading to LPRD symptoms such as chronic throat clearing and coughing [7]. Therefore, proton pump inhibitor (PPI) therapy is commonly recommended to improve laryngopharyngeal reflux (LPR)-related symptoms via its inhibitory effect on gastric acid. However, LPRD has poor responsiveness to PPI therapy compared with GERD [8]. Recent studies have attempted to prove the effectiveness of PPIs in treating LPRD and its associated comorbidities. Several placebo-controlled studies have demonstrated significant improvement in reflux laryngitis following PPI treatment [9,10,11]. In contrast, other studies have concluded that PPIs are not superior to placebo in treating suspected LPRD [12,13]. The adequate PPI dosage and treatment duration are controversial; however, several studies have demonstrated that prolonged treatment duration improves the effectiveness of PPIs in patients with LPRD [9,10,11]. In addition, twice-daily PPIs are more effective than once-daily PPIs in patients with LPRD by controlling both daytime and nocturnal esophageal acid exposure [14]. Therefore, twice-daily PPI therapy for 3–6 months is generally recommended for treating LPRD [15].

Recently, potassium-competitive acid blockers (P-CABs) have been suggested as an effective treatment for acid-related diseases [16]. Tegoprazan is a novel, potent, and highly selective P-CAB that was developed in Korea [17]. Unlike PPIs, which need chemical transformation into the active form and bind covalently to the gastric H^+^/K^+^-ATPase, tegoprazan inhibits H^+^/K^+^-ATPase reversibly in a K^+^-competitive manner without the need for any conversion. In addition, it blocks the active and inactive forms of proton pumps. Accordingly, tegoprazan inhibits gastric acid secretion with a rapid onset of action and leads to the prolonged control of gastric acidity [17,18]. In recent clinical trials, tegoprazan showed non-inferior efficacy in healing erosive esophagitis, tolerability similar to that of esomeprazole [19,20], and superior therapeutic efficacy compared with placebo in patients with non-erosive reflux disease (NERD) [21]. Thus, tegoprazan may have a better therapeutic effect in acid-related disorders, including LPRD. Therefore, this preliminary study aimed to evaluate whether an 8-week treatment with tegoprazan once daily significantly reduces LPRD symptoms and signs compared with placebo in patients with LPRD.

## 2. Materials and Methods

### 2.1. Study Population

This prospective, randomized, double-blind, placebo-controlled study was conducted at the Pusan National University Hospital (Busan, Republic of Korea) from October 2019 to December 2021. Patients aged ≥ 19 years who had exhibited at least one symptom of LPRD, including hoarseness, frequent throat clearing, a globus sensation, and persistent throat discomfort for at least 4 weeks were eligible for this study. All the patients completed the RSI questionnaire, consisting of a self-administered nine-item outcome instrument for diagnosing LPR, with the assistance of a trained nurse, and underwent a complete otolaryngologic examination by two otolaryngologists (B.J.L. and S.-C.S.). For inclusion in this study, their RSI score exceeded 13 [3], and their RFS score was >7 [4].

The exclusion criteria included the following: (1) viral or bacterial laryngopharyngitis at present; (2) history of a malignancy of the head and neck region, esophagus, and stomach; (3) previous radiotherapy or endotracheal intubation within 3 months; (4) previous anti-reflux or gastroesophageal surgery; (5) diagnosis of depression, anxiety, panic, somatoform, or other psychotic disorders; (6) anti-psychotics, anti-depressants, or anti-anxiety medications; (6) anti-reflux medication such as PPIs (within 4 weeks before screening), histamine receptor-2 blockers, antacids, or prokinetics (within 2 weeks before screening); (7) need for continuous therapy with non-steroidal anti-inflammatory drugs; (8) pregnant or breastfeeding women, and female patients who were not willing to use contraception for the duration of the clinical trial period; (9) abnormal laboratory test values at screening (blood urea nitrogen and serum creatine level >1.5 upper limit of normal [ULN]; serum levels of total bilirubin, alanine aminotransferase, aspartate aminotransferase, alkaline phosphatase, and gamma-glutamyl transferase >2 ULN); or (10) any other conditions or diseases considered not appropriate for this study by an investigator.

The trial was conducted following the guiding principles of Good Clinical Practice and the Declaration of Helsinki. The study protocol was approved by the Institutional Review Board of the Pusan National University Hospital (IRB number: H-1909-005-096). All the patients were provided with a detailed description of the study and signed an informed consent form before inclusion and initiation of the study procedures. This study was registered as a standard, randomized clinical trial (ClinicalTrials.gov: NCT05871398).

### 2.2. Randomization

During the screening visit, demographic characteristics, such as sex, age, body weight, height, and drinking and smoking habits, were collected. After obtaining informed consent, the eligible patients were randomized in a 1:1 ratio for treatment with tegoprazan 50 mg or a placebo once daily before bedtime. The patients and investigators were blinded to the medication randomization. Each patient visited the hospital at weeks 4 and 8 for the assessment of LPRD symptoms and laryngoscopic findings. Each patient completed the RSI questionnaire, and the RFS was re-administered by the same otolaryngologist during the two follow-up visits.

Compliance with medication and lifestyle modification were investigated by the study nurse during the follow-up visits. Compliance was determined based on the number of tablets remaining for each drug type at the follow-up visit. If drug compliance was ≥ 80%, the patient’s data were included in the final outcome measurements.

### 2.3. Study Assessments

#### 2.3.1. LPRD Symptom Assessment

The patients’ symptoms were assessed using the translated Korean version of the RSI questionnaire as a subjective measure. The RSI questionnaire is a validated nine-item, self-administered questionnaire for assessing the severity and responses to treatment of LPRD-associated symptoms (Supplemental Table S1) [3]. The individual components are scored from 0 (no problem) to 5 (severe problem), with a maximum total score of 45. An RSI score of >13 was considered abnormal [3].

#### 2.3.2. LPRD Sign Assessment

Laryngeal examinations were performed to document LPR signs, based on RFS as an objective measure. The RFS is a validated eight-item clinical severity rating scale, which was developed to quantify the degree of laryngeal involvement in LPRD during fiberoptic laryngoscopy (Supplemental Table S2) [4]. The scale has the most common laryngeal findings related to LPR. Scores range from 0 (no abnormal findings) to 26 (worst possible score), with scores > 7 considered abnormal [4].

#### 2.3.3. Study Efficacy

The primary efficacy endpoint was the complete resolution rate of LPRD symptoms after 8 weeks of medication. The secondary efficacy endpoints were as follows: (1) the complete resolution rate of LPRD symptoms after 4 weeks of medication; (2) mean reduction in the total RSI and RFS scores from baseline to weeks 4 and 8; and (3) mean change in the individual component scores of RSI and RFS from baseline to weeks 4 and 8. Complete resolution of LPRD symptoms was defined as when the total RSI score reached <13 points and ≥50% reduction of the initial RSI scores after 4 and 8 weeks of medication. The safety assessments of the medications included adverse events and adverse drug reactions, including their severity and duration.

### 2.4. Statistical Analysis

No previous study has investigated LPRD treatment using tegoprazan; hence, the sample size was determined based on a previous study that reported a typical LPR-associated sub-score would improve by at least one point in 65% of the PPI group and 40% of the placebo group [13]. The number of participants was calculated to be 74 in each group for detecting a significant association with a power of 80% and 5% two-sided type I error level (α = 0.05), assuming a 20% drop-out rate. We planned a preliminary study to investigate the feasibility of conducting a large-scale randomized clinical trial of tegoprazan for LPRD. Therefore, based on a pilot study by Julious [22], a sample size of 12 patients per treatment group was calculated, accounting for a 30% drop-out rate, and a final sample size of 18 patients per treatment group was calculated.

The patient data were subjected to two types of analyses: a full-analysis set (FAS) and a per-protocol set (PPS). The FAS analysis included all the participants who had data on the primary efficacy evaluation parameters after treatment with the study medications. The PPS analysis was focused on participants from the FAS analysis, with data indicating that they had completed the clinical trial according to the protocol. The safety analysis included all the data from randomly assigned participants treated with the study medications. The efficacy parameters are presented as frequency and proportion (with 95% confidence interval [CI]) in each group. Statistical analyses were performed using the t-test or Mann–Whitney *U*-test for continuous variables, and the χ^2^ test or Fisher’s exact test for categorical data. The changes in symptom scores with time were parametric in nature and analyzed using repeated measures analysis of covariance. Statistical analyses were performed using SAS (version 9.4, SAS Institute, Cary, NC, USA). Statistical significance was set at a two-sided *p*-value of ≤0.05.

## 3. Results

### 3.1. Patient Allocation and Baseline Clinicodemographic Characteristics

**Figure 1:**
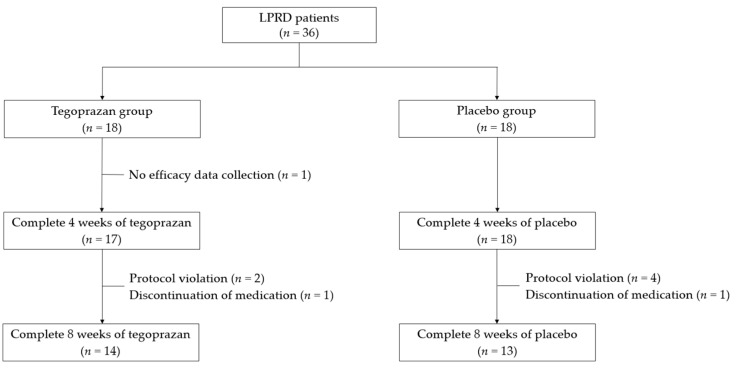
A flowchart of the patients included in the study.

In total, 36 patients were eligible for this study; 18 were randomized into the tegoprazan group and the other 18 into the placebo group. One patient in the tegoprazan group was excluded before study initiation, owing to the absence of efficacy data. Among the 35 patients, 3 in the tegoprazan group and 5 in the placebo group were excluded from the FAS analysis because of a violation of the visit day (tegoprazan group, *n* = 2; placebo group, *n* = 4) and discontinuation of the study medication (tegoprazan group, *n* = 1; placebo group, *n* = 1). Consequently, the data from 27 patients (tegoprazan group, *n* = 14; placebo group, *n* = 13) were used in the PPS analysis. The flowchart of patient progression throughout the study is illustrated in Figure 1.

The baseline clinicodemographic characteristics of the included 35 patients in both groups are presented in Table 1. There were no significant differences in sex, age, body mass index, alcohol consumption, or smoking status between the two groups. The drug compliance rates throughout the study period were 92.4% and 97.2% in the tegoprazan and placebo groups, respectively, and the drug complication rate did not differ significantly between the two groups (*p* = 0.162).

### 3.2. Efficacy Assessment

#### 3.2.1. Complete Resolution Rates of LPRD Symptoms after 4 and 8 Weeks of Medication

Based on the FAS analysis, the complete resolution rates of LPRD symptoms after 8 weeks of medication were 29.4% (5/17) and 27.8% (5/18) in the tegoprazan and placebo groups, respectively; there was no significant difference in the complete resolution rates of LPRD symptoms between the two groups (*p* = 1.000) (Table 2). In the PP analysis, the complete resolution rates of LPRD symptoms after 8 weeks of medication were 28.6% (4/14) and 38.5% (5/13) in the tegoprazan and placebo groups, respectively (*p* = 0.695).

Based on the FAS analysis, the complete resolution rates of LPRD symptoms after 4 weeks of medication were 11.8% (2/17) and 22.2% (4/18) in the tegoprazan and placebo groups, respectively, and there was no significant difference between the two groups (*p* = 0.658). In the PP analysis, the complete resolution rates of LPRD symptoms were 7.1% (1/14) and 30.8% (4/13) in the tegoprazan and placebo groups, respectively (*p* = 0.165).

#### 3.2.2. Changes in the Total RSI and RFS Scores from Baseline at 4 and 8 Weeks of Medication

(1) Changes in the total RSI scores after 4 and 8 weeks of medication

In the FAS analysis, both treatment groups achieved a significant decrease in the total RSI scores compared with the baseline after 4 weeks (tegoprazan group: 21.2 ± 6.2 at baseline to 16.8 ± 7.0 at week 4, *p* = 0.003, and placebo group: 22.1 ± 5.3 at baseline to 15.9 ± 7.6 at week 4, *p* < 0.001) and after 8 weeks of therapy (tegoprazan group: 21.2 ± 6.2 at baseline to 15.2 ± 7.8 at week 8, *p* = 0.002, and placebo group: 22.1 ± 5.3 at baseline to 16.2 ± 9.6 at week 8, *p* = 0.002) (Table 3). However, the mean change in the total RSI scores after 4 and 8 weeks of therapy was not significantly different between the tegoprazan and placebo groups (4 weeks, *p* = 0.394, and 8 weeks, *p* = 0.949). 

In the PP analysis, both treatment groups achieved a significant decrease in the total RSI scores compared with the baseline after 4 weeks (tegoprazan group: 20.8 ± 6.5 at baseline to 17.0 ± 7.3 at week 4, *p* = 0.019, and placebo group: 20.3 ± 4.9 at baseline to 14.1 ± 7.3 at week 4, *p* < 0.001) and after 8 weeks of therapy (tegoprazan group: 20.8 ± 6.5 at baseline to 15.1 ± 8.2 at week 8, *p* = 0.010, and placebo group: 20.3 ± 4.9 at baseline to 14.2 ± 9.8 at week 8, *p* = 0.008) (Table 4). However, the mean change in the total RSI scores after 4 and 8 weeks of therapy was also not significantly different between the two groups (4 weeks, *p* = 0.250, and 8 weeks, *p* = 0.886).

(2) Changes in total RFS scores after 4 and 8 weeks of medication

In the FAS analysis, the tegoprazan group showed a significant reduction in the total RFS scores after 4 and 8 weeks compared with the baseline (10.4 ± 2.4 at baseline to 8.5 ± 1.4 at week 4, *p* < 0.001, and 8.1 ± 1.4 at week 8, *p* < 0.001). The placebo group also showed a similar reduction in the total RFS scores (10.8 ± 1.8 at baseline to 9.1 ± 1.3 at week 4, *p* < 0.001, and 9.2 ± 1.8 at week 8, *p* < 0.001) (Table 3). There was no significant difference in the mean change in the total RFS scores after 4 and 8 weeks of therapy between the two groups (4 weeks, *p* = 0.279, and 8 weeks, *p* = 0.073). 

In the PP analysis, the tegoprazan group showed a significant reduction in the total RFS scores after 4 and 8 weeks compared with the baseline (10.7 ± 2.2 at baseline to 8.6 ± 1.4 at week 4, *p* < 0.001, and 8.2 ± 1.4 at week 8, *p* < 0.001). The placebo group also showed a similar reduction in the total RFS scores (10.5 ± 1.5 at baseline to 8.6 ± 1.0 at week 4, *p* < 0.001, and 9.0 ± 1.9 at week 8, *p* = 0.002) (Table 3). The mean change in the total RFS scores after 4 and 8 weeks of therapy was not statistically different between the two groups (4 weeks, *p* = 0.936, and 8 weeks, *p* = 0.219) (Table 4).

(3) Changes in the individual RSI items after 4 and 8 weeks of medication

Regarding the nine individual RSI items, the tegoprazan and placebo groups showed a significant reduction in hoarseness and throat sensation scores at weeks 4 and 8 compared with the baseline (Table 5). The placebo group also showed a significant reduction in the throat-clearing and throat mucus scores at weeks 4 and 8 compared with the baseline, and the tegoprazan group showed a significantly reduced throat-clearing score at week 8 compared with the baseline. Only the reduction in the throat mucus score at week 4 was significantly different between the two groups (−0.1 ± 1.5 vs. −1.1 ± 1.2, *p* = 0.032); however, the reduction in other individual RSI item scores did not differ significantly between the two groups at weeks 4 and 8.

(4) Changes in the individual RFS items after 4 and 8 weeks of medication

At week 4, only the tegoprazan group showed significantly reduced subglottic edema and vocal cord edema scores compared with the baseline (Table 5). The tegoprazan group also showed significantly reduced subglottic edema and vocal cord edema scores, and the placebo group showed significantly reduced vocal cord edema scores at week 8 compared with the baseline. The reduction in subglottic edema scores at week 8 was significantly higher in the tegoprazan group than in the placebo group (−0.8 ± 1.0 vs. 0.0 ± 1.2, *p* = 0.044); however, the reduction in other individual RFS item scores did not differ significantly between the two groups at weeks 4 and 8.

### 3.3. Adverse Events

Two patients in the tegoprazan group reported mild adverse events (Supplemental Table S3); one patient complained of back pain 2 weeks after treatment, and the other patient developed diarrhea 8 weeks after treatment. These adverse events were tolerable and both patients completed the study medication. No serious adverse drug reactions were observed in either group. 

## 4. Discussion

Considering the limited efficacy of PPIs in treating LPRD reported in previous studies, we prospectively evaluated the efficacy of tegoprazan, a potent acid-reducing agent, in patients with LPRD. An 8-week treatment with tegoprazan 50 mg daily improved LPRD symptoms and signs; however, there was no significant difference in the complete resolution rates of LPRD symptoms after 8 weeks of medication between the tegoprazan and placebo groups. Nonetheless, given the small number of included patients with LPRD, the results of our preliminary study suggest the potential role of tegoprazan in treating LPRD. To the best of our knowledge, this is the first study to assess the efficacy of P-CAB (tegoprazan) in patients with LPRD compared with placebo using internationally standardized LPR-related symptom and sign instruments (RSI and RFS). 

Tegoprazan has a rapid acid inhibition effect that is relatively long-lasting; the median time to achieve maximum drug concentration ranges from 1.4 to 1.8 h, and the plasma elimination half-life is approximately 4 h [23]. In addition, the pharmacodynamic and pharmacokinetic properties of tegoprazan are independent of food effects [24]. Therefore, tegoprazan is likely to have at least a therapeutic effect in acid-related disorders, such as GERD and peptic ulcer disease, similar to that of the PPIs. In 2018, tegoprazan was approved for treating reflux esophagitis in Korea [19], and it was recently approved for treating NERD [21], gastric ulcer [25], and *Helicobacter pylori* eradication [26].

In the present study, the complete resolution rate of LPRD symptoms after 8 weeks of medication in the tegoprazan group was 29.4%. This rate is lower than the complete resolution rates reported in previous studies (50.0% with lansoprazole 30 mg twice daily for 3 months [27], and 53% with rabeprazole 20 mg twice daily for 2 months [28]). Our relatively low response rate can be explained by the difference in treatment dose and duration and the difference in the clinical tools used to assess LPRD symptoms, which will be discussed in detail in the following paragraphs.

In the present study, acid-suppressive therapy with tegoprazan did not demonstrate superior benefits regarding complete resolution and improvement rates of LPRD symptoms and signs compared with placebo. These results are consistent with those of previous randomized controlled trials with PPIs [12,13,28]. In a study of 35 participants by Wo et al. [12], the total laryngeal symptom scores significantly improved compared with baseline in the two groups (pantoprazole 40 mg daily for 12 weeks vs. placebo); however, there was no significant difference in laryngeal symptom improvement rates between the two groups. Noordzij et al. also reported that omeprazole 40 mg twice daily for 2 months failed to demonstrate a superior therapeutic efficacy compared with placebo in patients with reflux laryngitis [13]. In contrast, esomeprazole 20 mg twice daily for 3 months showed significant improvement in total RSI and RFS compared with placebo, as reported by Reichel et al. [9]. Anzic et al. also reported that omeprazole 20 mg daily for 8 weeks significantly reduced RSI and RFS compared with placebo [11]. Lam et al. demonstrated a significantly decreased total RSI in a rabeprazole 20 mg twice-daily group compared with a placebo group; however, no significant difference in RFS was observed between the two groups [10]. Based on these studies, recent meta-analyses reported that PPI therapy could improve LPR symptoms significantly compared with placebo in patients with LPRD; however, there was no significant superiority in RFS compared with placebo [29,30].

These discrepancies can be explained as follows. First, different inclusion criteria were used. There is a lack of consensus regarding the diagnosis of LPRD, which is primarily based on subjective symptoms. The placebo effect is a well-documented phenomenon in clinical trials and occurs more commonly when the disorder is defined by subjective rather than objective findings [31]. Accordingly, it is not surprising that the treatment of LPRD is prone to the placebo effect. The objective findings of impedance-pH monitoring are helpful in diagnosing LPR; however, this is not always used in clinical practice, is not well tolerated, with a significant number of patients refusing impedance-pH monitoring, and is difficult to interpret [32]. Among other clinical tools, the RFS, which is a scoring system based on the endolaryngeal inflammatory findings suggestive of reflux, is the most frequently assessed objective tool for diagnosing LPR [4,33]. This tool also has some weaknesses in that 80% of healthy participants could have ≥1 sign of laryngeal irritation, including laryngeal erythema, diffuse laryngeal edema, and posterior commissure hypertrophy [33]; furthermore, in a study evaluating impedance–pH monitoring, GERD was diagnosed in <40% of patients with LPRD diagnosed by RSI and RFS, mainly owing to the low specificity of the laryngoscopic findings [34]. However, the RFS has been shown to have high reproducibility and reliability, and a patient with a score >7 has a 95% probability of presenting with LPR [4]. Several studies including patients based on RSI and RFS results have demonstrated significant improvements in treatment groups over placebo groups [9,10,11]. We also enrolled patients based on RSI and RFS results in this study; however, we could not prove the superiority of tegoprazan over placebo. 

Another reason could be the treatment duration and dose. The adequate treatment duration and PPI dosage are controversial; however, the recommended treatment in patients with LPRD is a twice-daily dosage of PPI therapy for 3–6 months [15]. Reichel et al. reported a significant placebo effect for LPRD, especially within the first 6 weeks of PPI treatment [9]. However, after 3 months of treatment, the PPI group showed more obvious symptom improvement than the placebo group; therefore, the researchers recommended that PPI treatment should continue for at least 3 months. A previous systematic review demonstrated no significant difference compared with the placebo when patients were treated for 4 weeks [35]. In the present study, the 8-week treatment duration might have been relatively short to demonstrate the superiority of tegoprazan over placebo. The change in total RFS after 8 weeks was not different between the tegoprazan and placebo groups; however, the reduction in subglottic edema score after 8 weeks of tegoprazan was more obvious than that of placebo (tegoprazan: −0.8 ± 1.0 vs. placebo: 0.0 ± 1.2, *p* = 0.044). Regarding PPI dosage, the main therapeutic scheme of LPRD consists of once-daily PPIs, once-daily high-dose PPIs, or twice-daily PPIs for a duration of 1–6 months [33]. Park et al. reported that twice-daily PPIs were more effective than once-daily PPIs in obtaining a clinical symptom response in cases of suspected LPRD, with a treatment duration of at least 2 months [14]. In the present study, we used tegoprazan 50 mg daily instead of tegoprazan 50 mg twice daily, because tegoprazan 50 mg daily is covered under Korean government insurance. The plasma elimination half-life of tegoprazan (4 h) is longer than that of PPIs (0.5–2 h) [36]; however, the daily dose of tegoprazan might not be adequate for controlling LPRD symptoms and signs. In a recent study on tegoprazan in patients with NERD, an increased dose of tegoprazan was more helpful for patients with NERD in whom typical GERD symptoms were not achieved with the standard dose [21]. Therefore, based on our results, tegoprazan 50 mg twice daily for a prolonged duration is strongly recommended for further studies on tegoprazan in patients with LPRD.

Finally, the RSI and RFS significantly improved in the tegoprazan and placebo groups in the present study. Before the trial, all the patients were educated by a study nurse on how to take the study medications and how to modify their lifestyles. Therefore, factors other than acid suppression by tegoprazan, such as lifestyle modification, may play a role in improving LPRD symptoms and signs. A previous randomized placebo-controlled trial demonstrated a significant improvement in LPR with behavioral changes [28]. 

The present study has some limitations. First, because we performed a pilot study to evaluate the efficacy of tegoprazan in LPRD treatment, the number of participants enrolled in the present study was small, increasing the likelihood of a type II error. In addition, we could not perform a sub-analysis to identify the specific characteristics of patients with LPRD who benefit from tegoprazan treatment, owing to the small number of included participants. Second, we did not perform 24 h impedance–pH monitoring, which is a more objective test for diagnosing LPRD. However, previous studies have shown no significant correlations between hypopharyngeal reflux episodes on impedance–pH monitoring and symptom improvement or LPRD symptoms [12,37].

In conclusion, tegoprazan effectively improved LPRD symptoms and signs; however, it was not superior to placebo in treating LPRD. Considering the potential effectiveness of tegoprazan as an acid-suppressing therapy and the possibility of a type II error due to a low number of included patients in this study, prospective, large-scale, multi-center studies with a higher dose of tegoprazan for a prolonged duration are required to elucidate the efficacy of tegoprazan in patients with LPRD. 

## Figures and Tables

**Table 1:** Baseline clinicodemographic characteristics of the study patients.

	Tegoprazan 50 mg	Placebo	*p*-Value
	(*n* = 17)	(*n* = 18)	
Age (years)	60.6 ± 12.2	58.4 ± 8.3	0.141 *
Sex			0.088 ^†^
Men	3 (17.6)	8 (44.4)	
Women	14 (82.4)	10 (55.6)	
Body mass index (kg/m^2^)	23.5 ± 3.1	25.0 ± 3.0	0.142 *
Waist (cm)	86.5 ± 6.8	89.3 ± 7.2	0.227 *
Alcohol consumption	3 (17.7)	6 (33.3)	0.443 ^†^
Smoking	0 (0.0)	1 (5.6)	1.000 ^†^
Reflux symptom index	21.2 ± 6.2	22.1 ± 5.3	0.633 *
Reflux finding score	10.4 ± 2.4	10.8 ± 1.8	0.502 *

Data are presented as mean ± standard deviation or number (%). * Two-sample *t*-test or Mann–Whitney *U*-test, ^†^ χ^2^ test, or Fisher’s exact test.

**Table 2:** Complete symptom resolution rates of laryngopharyngeal reflux symptoms after 4 and 8 weeks of medication.

	Week 4		*p*-Value	Week 8		*p*-Value
	Tegoprazan	Placebo		Tegoprazan	Placebo	
Full-analysis set						
Number of patients	17	18		17	18	
Complete resolution rate	2 (11.8)	4 (22.2)	0.658	5 (29.4)	5 (27.8)	1.000
Per-protocol set						
Number of patients	14	13		14	13	
Complete resolution rate	1 (7.1)	4 (30.8)	0.165	4 (28.6)	5 (38.5)	0.695

Data are presented as number (%).

**Table 3:** Changes in RSI and RFS from baseline after 4 and 8 weeks of medication: a full-analysis set.

	Group	Baseline	Week 4	*p*-Value *	Week 8	*p*-Value ^†^
RSI score	Tegoprazan (*n* = 17)	21.2 ± 6.2	16.8 ± 7.0	0.003	15.2 ± 7.8	0.002
	Placebo (*n* = 18)	22.1 ± 5.3	15.9 ± 7.6	<0.001	16.2 ± 9.6	0.002
	*p*-value ^‡^		0.394		0.949	
RFS score	Tegoprazan (*n* = 17)	10.4 ± 2.4	8.5 ± 1.4	<0.001	8.1 ± 1.4	<0.001
	Placebo (*n* = 18)	10.8 ± 1.8	9.1 ± 1.3	<0.001	9.2 ± 1.8	<0.001
	*p*-value ^§^		0.279		0.073	

RSI, reflux symptom index; RFS, reflux finding score. * Difference within treatment groups, Week 4—baseline, ANCOVA model with covariate as baseline RSI and RFS. ^†^ Difference within treatment groups, Week 8—baseline, ANCOVA model with covariate as baseline RSI and RFS. ^‡^ Difference between treatment groups, ANCOVA model with covariate as baseline RSI. ^§^ Difference between treatment groups, ANCOVA model with covariate as baseline RFS.

**Table 4:** Changes in RSI and RFS from baseline after 4 and 8 weeks of medication: a per-protocol set.

	Group	Baseline	Week 4	*p*-Value *	Week 8	*p*-Value ^†^
RSI score	Tegoprazan (*n* = 14)	20.8 ± 6.5	17.0 ± 7.3	0.019	15.1 ± 8.2	0.010
	Placebo (*n* = 13)	20.3 ± 4.9	14.1 ± 7.3	<0.001	14.2 ± 9.8	0.008
	*p*-value ^‡^		0.250		0.886	
RFS score	Tegoprazan (*n* = 14)	10.7 ± 2.2	8.6 ± 1.4	<0.001	8.2 ± 1.4	<0.001
	Placebo (*n* = 13)	10.5 ± 1.5	8.6 ± 1.0	<0.001	9.0 ± 1.9	0.002
	*p*-value ^§^		0.936		0.219	

RSI, reflux symptom index; RFS, reflux finding score. * Difference within treatment groups, week 4—baseline, ANCOVA model with baseline RSI and RFS as covariates. ^†^ Difference within treatment groups, week 8—baseline, ANCOVA model with baseline RSI and RFS as covariates. ^‡^ Difference between treatment groups, ANCOVA model with baseline RSI as covariates. ^§^ Difference between treatment groups, ANCOVA model with baseline RFS as covariates.

**Table 5:** Changes in individual RFS and RSI item scores from baseline after 4 and 8 weeks of medication.

	Tegoprazan (*p*-Value *)	Placebo (*p*-Value *)	Difference (*p*-Value ^†^)
Changes in score between baseline and week 4			
RSI score			
Hoarseness	−1.2 ± 1.9 (0.016)	−1.0 ± 1.4 (0.008)	−0.2 ± 1.7 (0.678)
Throat clearing	−0.5 ± 1.4 (0.146)	−0.9 ± 1.8 (0.049)	0.4 ± 1.6 (0.449)
Throat mucus	−0.1 ± 1.5 (0.750)	−1.1 ± 1.2 (0.002)	0.9 ± 1.4 (0.032)
Difficulty swallowing	0.1 ± 0.9 (0.789)	−0.2 ± 0.9 (0.500)	0.3 ± 0.9 (0.349)
Coughing after meals	−0.2 ± 1.0 (0.361)	−0.3 ± 1.5 (0.345)	0.1 ± 1.3 (0.959)
Breathing difficulty	−0.4 ± 0.9 (0.188)	−0.2 ± 1.4 (0.495)	−0.1 ± 1.1 (0.912)
Annoying cough	−0.5 ± 1.5 (0.257)	−0.3 ± 1.3 (0.491)	−0.2 ± 1.4 (0.622)
Throat sensation	−0.7 ± 0.9 (0.023)	−1.2 ± 1.3 (0.001)	0.6 ± 1.1 (0.190)
Heartburn	−0.9 ± 2.1 (0.058)	−0.9 ± 1.1 (0.002)	0.0 ± 1.7 (0.774)
RFS score			
Subglottic edema	−0.9 ± 1.0 (0.008)	−0.2 ± 1.4 (0.727)	−0.7 ± 1.2 (0.114)
Ventricular obliteration	0.0 ± 0.7 (1.000)	−0.4 ± 0.9 (0.125)	0.4 ± 0.8 (0.112)
Erythema	0.1 ± 0.5 (1.000)	−0.1 ± 0.5 (1.000)	0.2 ± 0.5 (0.176)
Vocal fold edema	−0.5 ± 0.6 (0.016)	−0.4 ± 0.7 (0.063)	−0.1 ± 0.7 (0.764)
Diffuse laryngeal edema	−0.2 ± 0.6 (0.250)	−0.1 ± 0.3 (0.500)	−0.1 ± 0.5 (0.568)
Posterior commissure hypertrophy	−0.2 ± 0.4 (0.125)	−0.1 ± 0.4 (1.000)	−0.2 ± 0.4 (0.235)
Granuloma/granulation tissue	0.0 ± 0.0	−0.1 ± 0.5 (1.000)	0.1 ± 0.3 (0.360)
Thick endolaryngeal mucus	−0.1 ± 1.3 (1.000)	−0.3 ± 1.2 (0.453)	0.2 ± 1.3 (0.637)
			
Changes in score between baseline and week 8			
RSI score			
Hoarseness	−1.1 ± 1.8 (0.019)	−1.0 ± 1.5 (0.013)	−0.1 ± 1.7 (0.834)
Throat clearing	−0.8 ± 1.6 (0.036)	−1.0 ± 1.4 (0.008)	0.2 ± 1.5 (0.643)
Throat mucus	−0.7 ± 1.8 (0.124)	−1.0 ± 1.2 (0.004)	0.3 ± 1.5 (0.398)
Difficulty swallowing	0.1 ± 1.1 (0.820)	0.1 ± 1.3 (1.000)	0.1 ± 1.2 (0.473)
Coughing after meals	−0.2 ± 0.8 (0.398)	−0.4 ± 1.3 (0.283)	0.2 ± 1.1 (0.439)
Breathing difficulties	−0.6 ± 1.2 (0.056)	−0.3 ± 1.1 (0.240)	−0.3 ± 1.2 (0.491)
Annoying cough	−0.7 ± 1.7 (0.127)	−0.5 ± 1.4 (0.125)	−0.2 ± 1.6 (0.568)
Throat sensations	−1.1 ± 1.4 (0.005)	−1.1 ± 1.0 (0.001)	−0.1 ± 1.2 (0.932)
Heartburn	−0.8 ± 2.1 (0.120)	−0.7 ± 2.1 (0.099)	−0.2 ± 2.1 (0.919)
RFS score			
Subglottic edema	−0.8 ± 1.0 (0.016)	0.0 ± 1.2 (1.000)	−0.8 ± 1.1 (0.044)
Ventricular	0.0 ± 1.0 (1.000)	−0.4 ± 0.9 (0.125)	0.4 ± 0.9 (0.184)
Erythema	0.0 ± 1.0 (1.000)	−0.1 ± 0.5 (1.000)	0.1 ± 0.8 (0.704)
Vocal fold edema	−0.6 ± 0.6 (0.004)	−0.4 ± 0.6 (0.031)	−0.2 ± 0.6 (0.290)
Diffuse laryngeal edema	−0.2 ± 0.7 (0.313)	−0.1 ± 0.3 (0.500)	−0.1 ± 0.5 (0.586)
Posterior commissure hypertrophy	−0.2 ± 0.6 (0.250)	−0.1 ± 0.3 (0.500)	−0.1 ± 0.5 (0.568)
Granuloma/granulation tissue	0.0 ± 0.0	−0.1 ± 0.5 (1.000)	0.1 ± 0.3 (0.360)
Thick endolaryngeal mucus	−0.4 ± 1.1 (0.375)	−0.3 ± 1.2 (0.453)	−0.0 ± 1.2 (1.000)

Data are presented as mean ± standard deviation. * Paired *t*-test or Wilcoxon signed rank test (within group). ^†^ Two-sample *t*-test or Mann–Whitney *U*-test (between groups).

## Data Availability

The data presented in this study are available on request from the corresponding authors.

## Supplementary Materials

The following supporting information can be downloaded at: https://www.mdpi.com/article/10.3390/jcm12196116/s1, Table S1: Reflux symptom index; Table S2: Reflux finding score; Table S3: Incidence of adverse drug reactions of the two medications.

## Abbreviations

CI: confidence interval; FAS: full-analysis set; GERD: gastroesophageal reflux disease; LPR: laryngopharyngeal reflux; LPRD: laryngopharyngeal reflux disease; NERD: non-erosive reflux disease; P-CAB: potassium-competitive acid blocker; PPI: proton pump inhibitor; PPS: per-protocol set; RFS: reflux finding score; RSI: reflux symptom index; ULN: upper limit of normal.
