# Supplementary material for: Efficacy of Tegoprazan in Patients with Laryngopharyngeal Reflux Disease: A Preliminary Feasibility Study
Source: J Clin Med. 2023 Sep 22;12(19):6116. doi: 10.3390/jcm12196116 (PMC10573336; doi:10.3390/jcm12196116)
Supplement: Supplementary file 1 [file jcm-12-06116-s001.zip › jcm-2573276-supplementary.pdf]

Supplemental Table 1. Reflux symptom index

Within the last month, how did the following problems affect you?	0 = no problem<br>5 = severe problem					
1. Hoarseness or a problem with your voice	0	1	2	3	4	5
2. Clearing your throat	0	1	2	3	4	5
3. Excess throat mucus or postnasal drip	0	1	2	3	4	5
4. Difficulty swallowing food, liquids, or pills	0	1	2	3	4	5
5. Coughing after you ate or after lying down	0	1	2	3	4	5
6. Breathing difficulties or choking episodes	0	1	2	3	4	5
7. Troublesome or annoying cough	0	1	2	3	4	5
8. Sensations of something sticking in your throat or a lump in your throat	0	1	2	3	4	5
9. Heartburn, chest pain, indigestion, or stomach acid coming up	0	1	2	3	4	5
Total						

Supplemental Table 2. Reflux finding score

Finding	Score
Subglottic edema	0 = absent
	2 = present
Ventricular obliteration	2 = partial
	4 = complete
Erythema/hyperemia	2 = arytenoids only
	4 = diffuse
Vocal fold edema	1 = mild
	2= moderate
	3 = severe
	4 = polypoid
Diffuse laryngeal edema	1 = mild
	2 = moderate
	3 = severe
	4 = obstructing
Posterior commissure hypertrophy	1 = mild
	2 = moderate
	3 = severe
	4 = obstructing
Granuloma/granulation	0 = absent
	2 = present
Thick endolaryngeal mucus/other	0 = absent
	2 = present
Total	

Supplemental Table 3. Incidence of adverse drug reactions of the two medications

	Tegoprazan 50 mg (*n* = 17)	Placebo (*n* = 18)
Gastrointestinal disorders		
Diarrhea	1 (5.9)	0 (0.0)
Musculoskeletal disorders		
Back pain	1 (5.9)	0 (0.0)
Total	2 (11.8)	0 (0.0)

Data are presented as number (%).
